# New biomarkers and multiplex tests for diagnosis of aggressive prostate cancer and therapy management

**DOI:** 10.3389/fonc.2025.1542511

**Published:** 2025-02-25

**Authors:** Milan Kral, Daniela Kurfurstova, Pavel Zemla, Martin Elias, Jan Bouchal

**Affiliations:** ^1^ Department of Urology, Medical Faculty, Palacký University and University Hospital Olomouc, Olomouc, Czechia; ^2^ Department of Clinical and Molecular Pathology, Medical Faculty, Palacký University and University Hospital Olomouc, Olomouc, Czechia; ^3^ Institute of Molecular and Translational Medicine, Medical Faculty, Palacký University, Olomouc, Czechia

**Keywords:** prostate, cancer, biomarker, test, treatment, prognosis

## Abstract

Despite improving diagnostic possibilities, the incidence of prostate cancer is increasing, but we are not able to reduce the mortality rate. While PSA, 4K score, PCA3 and other urinary markers, ExoDX, SelectMDX, Confirm MDx or MiPS tests are used to identify potential prostate cancer carriers, Decipher, Prolaris or Oncotype DX tests are used to assess the aggressiveness of proven cancer in order to stratify patients for early or delayed treatment. More modern forms of treatment for advanced disease include second-generation antiandrogens and PARP inhibitors. By assessing genetic mutations (e.g. BRCA1, BRCA2 genes, single nucleotide polymorphism) or the presence of splice variants of the androgen receptor (ARV7), we are able to identify patients in whom the planned treatment may be expected to be ineffective and thus choose other treatment modalities. In the present review article, we offer a comprehensive overview of current diagnostic tests that find application in the diagnosis of early and advanced prostate cancer.

## 1 Introduction

Globally, prostate cancer ranks second in the incidence of malignancies in men, with an estimated 1.4 million new cases worldwide in 2020 (1). Thus, prostate cancer accounts for 7.3% of all cancers. The mortality rate for prostate cancer was 375,000 cases (3.8%). Despite the steady increase in newly diagnosed diseases (usually in localized stages), mortality rates have not changed significantly due to early detection, maximal radical therapy and modern treatments for advanced and generalized cases. In the USA, although there has been a decrease in low-risk prostate cancer over two years, there has been an increase in metastatic stages (2). Although universal screening for prostate cancer is not recommended by the European and American Urological Associations (EAU, AUA), the increase in the detection of new cases is due both to screening programs in individual regions and to the increasing awareness of the disease among the general public, who are then directed towards active screening. The results of the largest international screening project, the European Randomized Study for Prostate Cancer (ERSPC), should be acknowledged and applied in clinical practice. The original data after 9 years of follow-up reported that 48 patients needed to be treated to prevent one death from prostate cancer. At a follow-up of 13 years, this number had already dropped to 26 men, and at a follow-up of 16 years, the number of patients had dropped even further to 18. This shows that the effect of screening on mortality reduction increases significantly with increasing follow-up time (3), see also **Table 1**. Despite the undeniable benefit on mortality reduction, it is generally recommended that the patient is properly informed about all the circumstances of screening and the risks involved before the actual screening. Overdiagnosis, i.e. overdiagnosis of cancer, is associated with universal screening, which does not always benefit the patient (see below). This is where certain biomarkers for prostate cancer or prostate tissue analysis play an important role. They allow to select patients at increased risk of carrying this cancer or to identify highly aggressive forms of cancer requiring early therapy. The purpose of the biomarkers and diagnostic steps used today is to optimally identify only those prostate cancers that are potentially life-threatening to patients (i.e. intermediate and high-risk cancers). Conversely, our goal is to avoid diagnosing low-risk tumors.

**Table 1:** Role of patients’ follow-up time on the effectiveness of prostate cancer screening (adapted from (3)).

Years of tracking	Number of patients required for screening	Number of patients required to be treated to prevent death from cancer
9	1410	48
11	979	35
13	781	27
16	570	18

## 2 The role of clinical examination in the diagnosis of prostate cancer

The initial diagnosis of prostate cancer in daily practice is based on digital rectal examination (DRE) and blood sampling for prostate specific antigen (PSA). While DRE has been used for many decades, the use of PSA dates to the 1990´s. DRE is characterized by suboptimal sensitivity and specificity values: although results vary from study to study, in the case of pathological findings on rectal examination according to Palmerol et al. the sensitivity was 44%, specificity 68%, positive predictive value 46% and negative predictive value 67% (4). However, it is important to note that, especially in today’s cohorts of men with prostate cancer indicated for radical treatment, most patients tend to be classified as stage T1c, i.e., a non-palpable tumor diagnosed because of PSA elevation or other biomarkers. Conversely, a suspicious palpation finding on the prostate significantly increases the rate of potential cancer.

## 3 Use of oncomarkers and tissue analysis

Oncomarkers are used in practice as prognostic and predictive. They differ in that a prognostic marker allows patients to be stratified according to the potential risk of adverse cancer outcomes during treatment and therefore to choose a more aggressive form of treatment. The predictive biomarker allows us to estimate the efficacy of the treatment and therefore to choose a specific type of therapy.

### 3.1 Types of prostate cancer markers

- Serum (PSA and derived parameters, PHI, 4K Score).
- Urinary (PCA3, Select MDx, ExoDX, MiPS).
- Tissue (Prolaris, Oncotype Dx, Confirm MDX, Decipher, Promark).

### 3.2 The purpose of using prostate cancer markers

- selection of patients who are potential cancer carriers (PSA and derived parameters), 4K score, PCA3 and other urinary markers, ExoDX, SelectMDX, Confirm MDx, MiPS and others).
- assessment of the aggressiveness of proven cancer to stratify patients for early or delayed treatment (Decipher, Prolaris or Oncotype DX).
- in predicting failure of newer forms of tailoring therapy (assessment of defects in DNA repair mechanisms and their significance in response to PARP inhibitors - splice variant of the androgen receptor gene - ARV7).

## 4 Types of tumor markers

### 4.1 Serum markers

#### 4.1.1 Prostate specific antigen and derived parameters

PSA (KLK3) is a serine protease classified as a human kallikrein. It is secreted by acinar cells of the prostate and its physiological role is to liquefy semen. Its level (ng/ml) is affected by a number of circumstances, including prostate cancer, benign prostatic hyperplasia (BPH), prostatitis or recent invasive investigations (urethrocystoscopy, bladder catheterization). Initially, the use of PSA was intended to monitor the progression of prostate cancer and subsequently its use was approved for the primary diagnosis of prostate cancer (5). Given the low reproducibility of rectal examination, PSA correlates better with the risk of prostate cancer, especially at stages when the tumor is undetectable by palpation. As shown in several multicenter studies, comparing groups of men enrolled and not enrolled in screening programs using PSA has repeatedly demonstrated that cancer-specific mortality decreases in the screened population. In addition, the number of patients requiring screening and treatment decreases with the length of follow-up (see **Table 1**). In the general population of men, PSA screening is recommended from age 50 to 75 years, with discontinuation recommended when life expectancy is less than 10 years. A special category is men in high-risk groups (e.g., African Americans, positive family history of prostate cancer or multiple malignancies in the family, positive testing for BRCA1 and BRCA2 mutations), where PSA testing is recommended starting at age 40 years. In other cases, PSA values follow an age-specific range (6, 7). Although PSA generally correlates with the risk of prostate cancer, as early as 2004, Thompson et al. (8) demonstrated in the Prostate Cancer Prevention Trial that there is essentially no normal PSA value. In fact, even at low PSA levels, prostate cancers, even high-grade or clinically advanced ones, have been detected, as described in some published case reports (9). Therefore, even in the case of so-called “normal PSA values” according to the age-specific range, this fact should be kept in mind. The risk of cancer according to ISUP grade ≥ 2 is 0.8% in patients with a PSA of 0-0.5 ng/ml and rises to 6.7% with a PSA between 3.1-4.0 ng/ml. Derived PSA parameters (density, velocity) further help to better stratify the risk of potentially present cancer, thus increasing the sensitivity and specificity of the test (10). The Prostate Health Index (PHI), Beckman Coulter, USA, is another biochemical parameter used in the prediction of prostate cancer. Its value is obtained by mathematical calculation from total and free PSA and also from the [-2]proPSA isoform according to the formula [(p2PSA/fPSA) × √tPSA]. Catalona (11) in a multicenter study evaluated PHI in patients with PSA ranging from 2-10 ng/ml. A cohort of 892 patients with increasing PHI showed a 4.7-fold increased risk of cancer and a 1.6-fold increased risk of high-grade cancer. PHI is a continuous variable and has the highest sensitivity and area under the curve (AUC) compared to PSA, fPSA and p2PSA. A value above 35-40 (depending on the specific laboratory) is used as the optimal cut-off. Compared with PSA alone, the benefit of PHI lies in better identification of high-grade cancers, as confirmed by other studies (12, 13). In addition to the diagnosis of prostate cancer itself, PHI has been used to predict worse pathological scores after radical prostatectomy (≥pT3 and/or GS ≥7), as demonstrated in a prospective study of 489 patients (14).

#### 4.1.2 4K score

The 4K Score test (OPKO Lab) involves the analysis of four serum biomarkers: total and free PSA, intact PSA and human kallikrein-related peptidase 2 (hK2). Using an algorithm that includes age, rectal findings and history of previous biopsy, a score is obtained with values ranging from 0-100. The primary purpose of use was to reduce overdiagnosis (and possibly overtreatment) in patients with low-risk prostate cancer. Its benefit lies in the prediction of high-grade prostate cancer (Gleason score ≥ 7) not only in the punch biopsy but in the radical prostatectomy specimen. Furthermore, the 4K Score allows the prediction of biological behavior, aggressiveness and the development of metastatic disease. This is because human kallikreins (PSA, i.e., hK3, and hK2, among others) are involved in the dedifferentiation of prostate cells, ensuring cell invasion and angiogenesis, among other things, which increases the risk of progression of the present cancer and metastasis. The primary validation of the assay was performed in a cohort of 740 patients from the Gothenburg arm of the European Randomized Study of Screening for Prostate Cancer (ERSPC), comprising patients with a PSA of 3-10 ng/ml who did not undergo screening (15). Analysis of data from this and other studies showed a 40-60% reduction in biopsy rates. The vast majority of patients with cancers that would have escaped diagnosis in this way were low risk. This brings economic and psychological savings as well as a reduction in the potential risks associated with repeat prostate biopsies (16, 17). The 4K Score can be used as a stand-alone test or used in conjunction with calculators (e.g. Rotterdam Prostate Cancer Risk Calculator - RPCRC). In a cohort of 2,872 men, it was shown that a model combining the 4K Score with a calculator had an even higher cancer detection rate and was also better able to identify patients with indolent prostate cancer (and thus spare them redundant biopsies) compared with the test alone (18).

### 4.2 Urinary markers

#### 4.2.1 PCA3

While PSA as a blood oncomarker has been used in clinical practice since the late 1980´s and early 1990´s, the search for another more specific oncomarker that could better predict the presence of (optimally significant) prostate cancer continued. In 1999 (19), they discovered another prostate oncomarker introduced into clinical practice, Prostate Cancer Gene 3 (PCA3). It is a long non-coding RNA whose product is not a protein. On the other hand, PCA3 has been shown to be involved in influencing the transcriptional activity of androgen receptor target genes and is a negative regulator of the tumor suppressor PRUNE2 (20). PCA3 is prostate-specific and is detected in varying amounts in localized and generalized forms of prostate cancer. Although PCA3 can be detected in both prostate tissue and blood serum, ultimately the analysis of PCA3 in the first portion of urine after prostate massage proved to be the easiest (and least invasive). During this procedure, sufficient prostatic epithelium is released into the prostatic urethra, where the cells are expelled by the urine stream and easily analyzed. The PCA3 (commercial name Progensa^®^) was marketed by GenProbe and was the first Food and Drug Administration (FDA) approved test to investigate prostate oncomarkers in urine. In clinical practice, the PCA3 score is used, which is given as the ratio of PCA3 mRNA copies to PSA mRNA copies x 1000. In prostate cancer tissue, the PCA3 mRNA level is elevated 60-100 times compared to normal tissue (21). By assessing the score values in healthy and prostate cancer patients, an arbitrary cutoff value for the PCA3 score of 35 was set as the optimal ratio of sensitivity to specificity. Similar to PSA, we encountered the fact that PCA3 scores are continuous and virtually no value may indicate the absence/presence of cancer. Thus, if we were to raise the cutoff value of the PCA3 score above the recommended 35, many patients with prostate cancer would miss out and conversely, if we lowered the score, we would increase the number of negative findings. It should be noted that several studies evaluating cancer detection rates have been conducted in the years following market introduction, with conclusions recommending even lowering the cut-off score to 25, among others, to identify insignificant prostate cancer (22, 23). In clinical practice, at the time of its introduction, PCA3 did not serve as a key marker of prostate cancer, but usually as an additional ancillary marker in the context of diagnostic confusion, e.g., when considering primary prostate biopsy or rebiopsy (especially at a time when magnetic resonance imaging (MRI) of the prostate was not as widespread and available in daily practice as it is today). Although the PCA3 test has been commercially available, its widespread use in screening is not recommended and thus plays a more supportive role when considering indications for prostate rebiopsy. On the other hand, the use of PCA3 has found its application in the research plans of some research centers, e.g., in the form of multiplex urinalysis (24, 25).

#### 4.2.2 Fusion genes in prostate cancer diagnosis

Genetic changes, usually translocations, are commonly detected in hematological malignancies and sarcomas. This results in changes in the regulation of oncogene expression. One of the first fusion genes in solid tumors was identified in 2005 (26). It was a fusion of the TMPRSS2 gene to the transcription factors ERG or ETV1 (also e.g. ETV4, ETV5). TMPRSS2 is a serine transmembrane protease expressed in the prostatic epithelium, the precise role of which is still unknown. However, we know that it is involved in a number of physiological and pathological processes in the prostate. Transcription factors (e.g. oncogene ERG etc.) respond to mitogenic stimuli and can lead to the activation of genes that induce carcinogenesis. It is the presence of these genetic changes (TMPRSS2/ERG fusion) that has been demonstrated in more aggressive types of carcinomas. In fact, these genes have been associated in several studies with more advanced stages, lymphadenopathy or biochemical failure after radical treatment; on the other hand, this association has not been clearly demonstrated by some studies (27–29). The TMPRSS2/ERG fusion gene can be detected not only in prostate tissue but also in urine. A study analyzing the presence of RNA of this fusion gene by RT-PCR confirmed that if TMPRSS2/ERG was present in urine, subsequent fluorescence *in situ* hybridization (FISH) confirmed the presence of the fusion gene in prostate tissue. Since TMPRSS2/ERG is present in more than 50% of prostate cancer tumors, its presence in urine may predict the presence of cancer (30). For this reason, this fusion gene has become another potential biomarker used for the diagnosis of prostate cancer (31), not only as a single parameter but also in combination with others, such as PCA3. One of the multiparametric tests, the MiPS (Michigan Prostate Score) includes analysis of PCA3, TMPRSS2/ERG and serum PSA to improve detection of high-grade cancers (32). MiPS2 is then a modified version including 18 analyzed genes that further refines the prediction of high-grade cancers and, importantly, reduces the need for diagnostic steps (imaging or biopsy) in 35-51% of patients (33).

#### 4.2.3 Select MDx

Another test using urine after prostate massage is the Select MDx. This is an examination of the mRNA of three genes in the urine (DLX, HOXC6 and TDRD1). Its benefit is not only to improve the prediction of the presence of prostate cancer, but to assess its aggressiveness, i.e. the clinical significance of the cancer (34). The positiveness of the test is associated with a higher risk of carrying high-grade cancer (up to 98% negative predictive value for a Gleason score ≥ 7) and, conversely, a low test score is highly likely to exclude high-risk cancer. Thus, low scores allow to reduce the indication for prostate biopsy without compromising the patient (underdiagnosis) (35). The yield of Select MDx can be further potentiated by combining it with prostate MRI evaluation. Hendriks et al. tested the yield of Select MDx in biopsy-naive men at risk of prostate cancer with and without prostate MRI. In the Select MDx test alone, 38% of patients were spared biopsy (10% of high-grade cancers were undetected), but in the combination of Select MDx and prostate MRI, 49% of patients avoided biopsy (and 4.9% of high-grade cancers were undetected) (36).

#### 4.2.4 ExoDx prostate test

The above tests (PCA3, TMPRSS2/ERG, MiPS and Select MDx) used the first portion of urine obtained after digital rectal examination for analysis. The ExoDx test uses a spontaneously voided first portion of urine without the need for a prior rectal examination. ExoDx analyses exosomal RNA of three genes that are elevated in high-grade prostate cancer: ERG, PCA3 and SPDEF. By analyzing data from 1000 men over 50 years of age with a PSA of 2-10 ng/ml referred for prostate biopsy, the so-called EPI (ExoDx Prostate IntelliScore) was determined to be 0-100 using a special algorithm. The threshold was arbitrarily set at 15.6 for low risk (EPI below 15.6) and high risk (EPI above 15.6) patients. The ExoDx test can be used as a stand-alone test or in combination with calculators (ERSPC Risk Calculator and PCPT Risk Calculator). This test can save more than 25% of men from having a prostate biopsy. The high sensitivity (92%), negative (91%) and positive (36%) predictive values have been subsequently confirmed in a number of other studies (37, 38). ExoDx test scores have also been correlated with postoperative histological findings from radical prostatectomies, suggesting the importance of more accurate and safer patient selection for active surveillance (39). Although DRE is by no means a complicated or poorly tolerated examination, the role of ExoDx arises, for example, in patients who have undergone a procedure in the rectal/anal region (e.g., proctocolectomy), cannot undergo prostate MRI for various reasons, and at the same time are suspected for the possible presence of significant prostate cancer. Due to the availability of the so-called home kit, the ExoDx Prostate Test has gained importance, among others, during the COVID-19 pandemic.

### 4.3 Prostate biopsy and tissue analysis

Prostate biopsy plays a key role in diagnosis. The indication for prostate biopsy is PSA elevation or suspicious rectal findings. In the case of PSA as an indication criterion, it must be said that many departments may differ in the PSA value they consider as a “trigger” for biopsy. If we take a PSA range of 2.5-4.0 ng/ml as an indication, the probability of prostate cancer on biopsy is approximately 25% (40). If we reduce this value below 2.5 ng/ml, fewer cancers will miss diagnosis, but at the cost of an excess of negative biopsies. Conversely, if we were to increase the PSA cut-off to, for example, 6-8 ng/ml, the number of negative biopsies would decrease, but at the risk of missing a relatively high number of potentially significant prostate cancers in the PSA range of 4-6 ng/ml. Great discussions have long been held on the number of prostate samples to be taken. Prostate biopsies were first sextan, then octant, replaced by multiple (10-14 samples) to saturation biopsy (20 or more samples) (41). Up to a certain number of samples, cancer detection increased, at the cost of increased discomfort and potential risks (urinary infection, hematuria). Thus, as noted above, the focus has shifted to how to refine the indication and diagnostic yield of biopsies. In other words, how to more appropriately refer patients who are likely to be carriers of high-risk cancer and, conversely, spare biopsies for those men who do not have prostate cancer (or are carriers of insignificant cancer). In the era of modern imaging (MRI), there is a clear shift away from saturation biopsies, and we are seeing a trend toward targeted biopsies (42), whether by transrectal or transperineal navigated approaches (43). In addition, given the common multifocality of tumor foci in the prostate, biopsies are being targeted to sites previously considered unlikely to be affected. For example, according to the work of Kudláčková et al. (44), it has been shown that up to 37% of carcinomas are present in the anterior zone of the prostate, and these tumors tend to be even significant and thus potentially life-threatening in many cases. Therefore, there is an increasing emphasis on tissue analysis that can identify the presence of an aggressive tumor in another prostate site even in the absence of an aggressive tumor on biopsy. This is where genetic analyses of prostate tissue are gaining ground. Data outputs from 1,561,203 US patients with prostate cancer treated between 2011 and 2021 showed that 20,748 patients, or 1.3% of the entire cohort, underwent some form of tissue genetic testing to better stratify cancer risk (Oncotype DX, Prolaris, Decipher, ProMark) (45). From pathologist´s point of view, the histology specimen is assessed to evaluate presence of different pathological prognostic parameters. Apart from most used Gleason score, other predictive factors are used, either in daily practice or as an investigational marker (PSA, PSMA, Ki-67, PD-L1, CDK19, E-catherin and many more) (46).

#### 4.3.1 Confirm MDX

Despite navigating the biopsy to suspicious foci (MRI, transrectal US), in many cases the biopsy is a false negative because the carcinoma foci were not detected in the biopsy core. Here, the so-called epigenetic analysis of the tissue may be advantageous as it assesses the so-called “halo” effect in the vicinity of the tumor tissue. Confirm MDx from MDxHealth, a test that analyses the hypermethylation of three genes that are associated with the presence of prostate cancer, can be used for this purpose (47). These genes are APC (Adenomatous Polyposis Coli), RASSF (Ras association domain family member 1) and GSTP1 (Glutathione S-Transferase Pi 1). In the case of a positive Confirm MDx test in a patient with a negative prostate biopsy, there is a high risk of prostate cancer. In the MATLOC study, Stewart et al. included 498 patients with a previous negative prostate biopsy. The negative predictive value (NPV) of the test was 90% and this test was found to be an independent predictor of cancer (48). This was validated in the subsequent DOCUMENT study (49). 350 men with a previous negative prostate biopsy underwent epigenetic analysis and rebiopsy over the next 2 years, and the test achieved a negative predictive value (NPV) of 88% (95% CI 85-91). In the next step, van Neste et al. created a so-called EpiScore using a special algorithm to better identify methylation-positive patients. This resulted in an NPV of 96%) (50), higher than other variables (PSA, atypia or high-grade PIN in previous biopsy, abnormal DRE or age). Due to cost, this test is not used before primary biopsy but only when rebiopsy is considered and Confirm MDx is also incorporated in the NCCN guidelines for this indication.

#### 4.3.2 Decipher (genomic classifier)

It is a multitest analyzing 22 RNA markers in prostate tissue. The profiles of two groups of patients were compared - men after radical prostatectomies who had progression or metastasis versus men with postoperative favorable parameters. The expression of these markers was determined using a score of 0-1. The results of the primary studies were subsequently validated by additional work. The risk of local cancer progression, biochemical recurrence and metastasis increased with increasing score. In 2021, a paper was published determining the GC score in a cohort of 352 male radical prostatectomy specimens with a median follow-up of 13 years. Patients were analyzed for prostate tissue with the least favorable histologic type. Considering other variables (age, race, PSA, Gleason score, disease stage, surgical margins), the GC score in the prostate was shown to be associated with the risk of distant metastasis and cancer-specific mortality (51), with the risk increasing with each 0.1 increase in the score. Although a large proportion of studies have focused on postoperative risk assessment and the indication/timing of adjuvant or salvage therapy (androgen deprivation or radiation) (52), Decipher is also used in patients with low to intermediate-risk prostate cancer who are considered for active surveillance. For this reason, according to ASCO guidelines (American Society of Clinical Oncologists), it has been included in the panel of tests potentially recommended to better stratify low-risk patients (53). One option is to categorize patients into low (<0.4), intermediate (0.4-0.6) and high risk (≥0.6) groups according to GC score. Spratt et al. (54) demonstrated 10year cumulative incidences of metastasis according to a given category of 5.5%, 15.0%, and 26.7%, respectively and allowed the AUC for this test to be increased to 0.81. Combining postoperative adverse parameters (pT3b/T4 disease, pathological Gleason score 8-10, lymph node invasion) and GC values > 0.6, Dalela et al. created an early model (values 1-4) to indicate adjuvant radiotherapy. Patients with a score of 2 or higher had a significantly lower risk of 10year clinical recurrence if adjuvant RT was indicated (10.1% in adjuvant setting vs. 42.1% in observation group) (55). A recent large meta-analysis (30 407 patients) in 42 studies confirmed that GC correlates as a prognostic marker with worse pathological findings, risk of biochemical relapse, metastasis, tumor-specific and overall survival. Crucially for clinical practice, according to five prospective randomized trials of 4331 patients (both assigned to active surveillance and active treatment), knowledge of GC outcome led to a change in treatment strategy. The number needed to test (NNT) was 9 in the active surveillance arm and 1.5-4 in the post-prostatectomy arm (56).

#### 4.3.3 Prolaris (cell-cycle progression score)

Another genetic test based on tissue analysis is Myriad’s Prolaris. Samples for analysis can be used from biopsy, transurethral resection or radical prostatectomy. It uses RT-PCR to evaluate 31 genes involved in the cell cycle and 15 housekeeping genes (57). Similar to the Decipher test, the cell-cycle progression score (CCP score) is evaluated to determine the risk of biochemical recurrence or metastasis after primary treatment. In most cases, the score ranges from 1-11, with increasing scores indicating a higher risk of more aggressive cancer behavior. In a Chinese study including 100 high-risk patients (pT3 or with positive surgical margins), CCP scores were determined as low risk (<0), intermediate risk (0-1), and high risk (>1). The 5-year survival without biochemical recurrence was 89% for low risk, 39% for intermediate risk, and only 13% for high risk (58). A more extensive study on a cohort of 236 patients after radical prostatectomy, evaluated as low risk according to NCCN recommendations, was published by Tosoian (59). Taking into account the CAPRA score (Cancer of the Prostate Risk Assessment), the probabilities of 5year biochemical recurrence-free survival for CCP score groups <0, (0-1) and >1 were found to be 89.2%, 80.4% and 64.7% respectively. Thus, by combining analysis of genetic testing and clinicopathological parameters (PSA, stage, Gleason score), potential candidates for active surveillance can be better and more safely stratified.

#### 4.3.4 ProMark

The purpose of this test was to create a tool that could predict favorable and unfavorable pathology from the biopsy itself. In clinical practice, it is evident that both the urologist (sampling error, so-called undersampling during biopsy) and the pathologist (interindividual variability of histology assessment) are involved in inappropriate treatment strategies for prostate cancer. The ProMark test is an automated imaging method using immunofluorescence *in situ*. It was originally based on 12 biomarkers analyzed in tumor tissue in one patient at a time, in a low-grade tumor (Gleason 3 + 3) and in a high-grade tumor (Gleason 4 + 4) (60). Subsequently, the set of tested proteins was narrowed down by analyzing 8 tumor progression-related biomarkers. Their selection was made on a cohort of 381 patients and subsequently validated on a cohort of 276 patients (61). The analysis yielded a ProMark score of 0-100, where the risk of aggressive tumor progression increases with increasing value. The test result can be applied in patients under active surveillance or in the prediction of disease progression after radical treatment (45).

#### 4.3.5 Oncotype DX (genomic prostate score)

Like Prolaris, Myriad’s Oncotype DX is based on analysis of prostate tissue. Even a sample size below 1 mm is sufficient, so carcinoma tissue from prostate biopsies can be used. The primary study analyzed 732 candidate genes, from which 81 genes were selected for further evaluation (these were all examined in the biopsy and radical prostatectomy sample arms). Finally, 12 genes involved in prostate carcinogenesis and 5 control genes were selected (62). Using an algorithm, the Genomic Prostate Score was determined to range from 0-100, with higher values increasing the aggressiveness and risk of cancer (63, 64). Covas Moschovas performed a retrospective analysis on 749 patients referred to radical prostatectomy. Multivariate analysis evaluating the odds ratio per 20point change of GPC (but also GPS quartile) showed a correlation with the frequency of local disease progression (extraprostatic spread or vesicle invasion) (65). In clinical practice, GPS is used in the decision-making process after prostate biopsy in patients with low and very low-risk prostate cancer (to indicate radical treatment vs. active surveillance).

#### 4.3.6 ARV7

The androgen receptor (AR) plays a key role in the tumor biology of prostate cancer. Under normal conditions, it is activated by androgens (testosterone, dihydrotestosterone) via ligand-receptor binding and this pathway is also used for systemic treatment of advanced and generalized stages, i.e. androgen deprivation (ADT, surgical or medical castration). When tumor progression occurs despite the initial androgen deprivation therapy, alternative pathways of androgen receptor activation are applied. These include, among others, amplification of the AR (i.e. increased sensitivity to even small amounts of androgens) or its mutation. These may lead to activation of the receptor by ligands other than androgens, e.g. other steroid hormones or even antiandrogens, which are otherwise used in many cases for actual hormone therapy. It is the role of antiandrogens as stimulants of tumor progression that has been confirmed in the context of the so-called antiandrogen withdrawal syndrome, a situation in which there is a favorable decline in PSA after withdrawal of first -generation antiandrogens for continued biochemical progression (66). Since the second-generation antiandrogens (enzalutamide, abiraterone) are one of the key agents in the treatment of castration-resistant prostate cancer, understanding the mechanism of their non/effect is critical. Mutations can result in the formation of so-called androgen receptor variants (ARVs), of which there are approximately 20. The formation of variant ARs leads to escape from the therapeutic mechanism and the detection of ARV7 specifically is associated with poor response to enzalutamide and abiraterone treatment. Detection of ARV7 mRNA is performed in circulating tumor cells (CTCs). Their role has been demonstrated across cancer and allows monitoring of disease progression and treatment effect (67, 68). By analyzing these cells in prostate cancer, patients can be classified as ARV7 positive and negative. In a cohort of 31 patients treated with enzalutamide and 31 treated with abiraterone, Antonarakis monitored the response to treatment in cases of ARV7 positivity and negativity. ARV7-negative patients treated with enzalutamide had a significantly better response in terms of PSA decline (P=0.004), duration of survival without PSA progression (P<0.001), time to clinical or radiological progression (P<0.001), and overall survival (P=0.Similar outcomes were obtained in the abiraterone arm (69). The same findings were confirmed in a cohort of 118 patients with metastatic castration-resistant prostate cancer treated with abiraterone or enzalutamide. ARV7-positive patients had worse/no response in PSA change (0-11%) soft tissue metastases (0-6%) (70). In contrast, ARV7-positive patients appear to benefit from chemotherapy using taxanes (71).

#### 4.3.7 Other genetic testing options

Prostate cancer is an example of a malignancy whose occurrence can be not only sporadic but also genetically determined. This is based on several epidemiological studies that have clearly demonstrated a familial burden not only in the presence of prostate cancer in the direct vertical or horizontal line, but also in related cancers (breast, ovary, colorectum, pancreas). Patients at increased risk are those with 2 or more members with prostate or breast cancer, as well as prostate, breast, colorectal or pancreatic cancer in the younger age group (under 50 years), in high-grade prostate cancer, especially cribriform or intraductal type, and in descendants of Jews of the Ashkenazi tribe. Genetic testing of these patients is also in the NCCN guidelines. Mutations in the so-called DNA repair genes, which provide natural genome protection, can be demonstrated. Such genes include BRCA1, BRCA2, ATM, CHEK2, and such mutated genes are passed on to subsequent family lines with a logically increasing risk of malignancy (51, 72, 73). It is well established that somatic or germline mutations are present in 20-25% of cases of metastatic castration-resistant prostate cancer. On the other hand, the demonstration of such aberrations is associated with the prediction of the efficacy of therapy for metastatic prostate cancer. For example, its carriers are more likely to have a positive response to treatment with second-generation antiandrogens or PARP (poly-adenosine diphosphate-ribose polymerase) inhibitors. PARP inhibitors, such as olaparib, have shown very good efficacy in the treatment of patients with BRCA1/2 or other mutations (74, 75) and have been implemented in the recommendations of many cancer societies even outside studies. Another option for genetic analysis is the determination of single nucleotide polymorphism (SNP). This is a genetic change in a single nucleotide (A, G, C, T) that leads to increased susceptibility to malignancy. In the case of prostate cancer, some of the most significant candidate genes or polymorphisms are MSR1, RNAseL and ELAC2. Specifically, the SNP for the enzyme RNAseL is probably of greatest importance, as this mutation leads to disruption of cell cycle regulation, differentiation and apoptosis (76, 77).

## 5 Summary of diagnostic options for prostate cancer: which test, for whom and when

In the diagnosis, prognosis assessment, and treatment planning of prostate cancer, a variety of tests are available to guide decision-making and patient management (**Table 2**). Primary diagnostics rely on key methods such as PSA level assessment and its derived parameters (PHI or 4K Score). These remain fundamental and will continue to be essential for the foreseeable future, alongside prostate MRI. For cases with persistent PSA elevation or disease progression, additional tests such as ExoDx, Confirm MDx, Select MDx, PCA3, TMPRSS2/ERG, or MiPS2 can be utilized. The availability and use of these tests often depend on factors such as insurance coverage and regional healthcare logistics, as transporting biological samples can be time-consuming, complex, or even unfeasible in some cases. Another critical aspect, particularly from the patient’s perspective, is the evaluation of prostate cancer aggressiveness once a diagnosis has been established. With the increasing number of patients eligible for active surveillance, the goal is to minimize the side effects of cancer treatment while achieving cost savings for healthcare systems. However, the challenge remains in preventing understaging, undergrading, and undertreatment, which could lead to serious clinical consequences. At this stage, urine-based markers (e.g., ExoDx, Select MDx) and especially tissue-based markers (Prolaris, Oncotype DX, or ProMark) play a crucial role in accurately assessing the risk of high-grade prostate cancer. These tests help refine patient selection for active surveillance, increasing both psychological comfort and clinical safety while reducing the risk of disease progression. For example, a study by Eure et al. (78) involving 505 low-risk prostate cancer patients found that incorporating the Decipher test into the decision-making process increased the number of patients enrolled in active surveillance by 23%, while also improving adherence to the protocol after one year of follow-up. Similarly, a prospective study conducted in Veterans Affairs medical centers demonstrated that patients who underwent Oncotype DX testing were significantly more likely to opt for active surveillance compared to those who were not tested (79). Beyond active surveillance, genomic risk assessment also informs the indication and timing of adjuvant or salvage therapy following radical treatment. In the multicenter PRO-IMPACT study, which included 246 patients considered for adjuvant (ART) or salvage (SRT) radiotherapy, treatment recommendations were significantly altered following Decipher test results. Specifically, ART recommendations increased from 12% to 17%, while SRT recommendations decreased from 40% to 30%. Importantly, patients also reported reduced anxiety regarding treatment decisions (80). In another study, Shore et al. (81) assessed the CCP score (Prolaris) in a cohort of 1,206 newly diagnosed prostate cancer patients. Their findings revealed that Prolaris testing led to a change in treatment decisions in 47.8% of cases, with 72.1% of these changes resulting in reduced treatment intensity and 26.9% leading to more aggressive interventions. Moreover, Prolaris significantly influenced therapeutic choices (radical treatment vs. observation) across all clinical risk categories (p=0.0002). In a prospective study Hu et al. (82) investigated the impact of gene expression classifiers (GECs) on treatment decisions in newly diagnosed prostate cancer patients. Among 3,966 patients from the Michigan Urological Surgery Improvement Collaborative registry, 747 (18.8%) underwent GEC testing (Decipher, Oncotype DX Prostate, or Prolaris). The study revealed substantial variability in test usage among different medical practices (0–93%). Patients with lower PSA levels, favorable clinical staging, and lower Gleason scores were more likely to undergo gene testing. Additionally, among low-risk patients, those with high classifier scores were significantly more likely to pursue active surveillance (75.9%) compared to those below the test threshold (46.2%). For advanced and metastatic prostate cancer, analysis of circulating tumor cells and circulating mRNA (e.g., ARV7 mRNA) as well as gene mutation testing (e.g., BRCA1 and BRCA2) can help predict treatment response, particularly for second-generation antiandrogens and PARP inhibitors, and provide valuable prognostic insights.

**Table 2:** Prostate cancer biomarkers: their clinical utility, advantages and limitations.

test	type of biomarker/test	biomarker	target patients group	role of the test	clinical advantage of the test	test disadvantage/ limitation
PSA	blood (serum)	PSA	50-75 years of age 40 years of age in risk (family history/Afroamericans) life expectancy based	primary biopsy/rebiopsy indication cancer significance stratification monitoring of cancer therapy	prize and availability	suboptimal sensitivity and specificity
PHI	blood (serum)	PSA, free PSA, [-2]proPSA isoform	50-75 years of age 40 years of age in risk (family history/Afroamericans) PSA 2-10 ng/ml	primary biopsy/rebiopsy indication cancer significance stratification	higher sensitivity and specificity over PSA in cancer detection ability to detect high grade cancers unnecessary biopsy reduction	imaging methods usually necessary in combination before re/biopsy indication
4K score	blood (serum)	PSA, free PSA, intact PSA, hK2 in combionation with DRE, age, previous biopsy result	PSA elevation	primary biopsy/rebiopsy indication prediction of clinically significant prostate cancer	effective in high grade cancers unnecessary biopsy reduction	may be of limited availability higher cost
Progensa (PCA3)	urine	PCA3 (Prostate Cancer Gen 3) non coding mRNA overexpressed in > 90 % cancers	PSA elevation	rebiopsy indication	predicts biopsy outcome better than PSA optimal to combine with other test/imaging methods	aggressive cancers may escape low sensitivity in lower stages optimal PCA3 score cut-off is controversial
TMPRSS2-ERG	urine, tissue	fusion genes TMPRSS2-ERG/ETV single or multiparametric test	PSA elevation patients at risk	rebiopsy indication prediction of clinically significant prostate cancer	may improve identification of high risk cancers better outcome in multiparametric tests	detection/expression may vary due to tumor heterogenity
MiPScore	urine	PCA3 (Prostate Cancer Gen 3) in combination with PSA and fusion genes TMPRSS2-ERG/ETV	PSA elevation patients at risk	prediction of clinically significant prostate cancer	may improve identification of high risk cancers better outcome than PCA3 alone unnecessary biopsy reduction	lower effectivity in lower grade tumors
Select MDx	urine	mRNA of genes HOXC6, TDRD1 and DLX1(overexpressed in high risk cancers)	PSA elevation patients at risk	primary biopsy/rebiopsy indication	may improve identification of high risk cancers unnecessary biopsy reduction low score highly excludes high risk cancers lowers risk of underdiagnosis combination with imaging improves outcome	may be of limited availability higher cost
ExoDX (IntelliScore)	urine	analyses exosomal RNA of PCA3, ERG and SPEDF	PSA 2-10 ng/ml patients at risk	primary biopsy/rebiopsy indication risk stratification	DRE not needed (helpful when DRE in impossible) unnecessary biopsy reduction safer selection candidates for active surveillance available as home kit test	may be of limited availability higher cost
Confirm MDX (EpiScore)	tissue	DNA methylation assay analysing APC, RASSF and GSTP1)	patients at risk after previous negative biopsy	rebiopsy indication	epigenetic analysis (of so called "halo" effect in cancer negative tissue high negative predictive value	may be of limited availability higher cost
Decipher (Genomic Classifier, GC Score)	tissue	RNA analysis of 22 genes GC score	patients with proven prostate cancer in biopsy or post-prostatectomy tissue	prediction of clinically significant prostate cancer stratification of indication and	may improve identification of high risk cancers may change treatment decision (under/overtreatment reduction) indication and timing of adjuvant treatment	may be of limited availability higher cost
Prolaris (Cell-cycle progression score)	tissue	PCR analysis of 31 cell-cycle genes and 15 house-keeping genes	patients with proven prostate cancer in biopsy or post-prostatectomy tissue	assessment of cancer aggressivness	determines the risk of biochemical recurrence or metastasis after primary treatment	may be of limited availability higher cost
ProMark (ProMark Score)	tissue	automated imaging method using FISH	patients with proven prostate cancer in biopsy or post-prostatectomy tissue	assessment of cancer aggressivness	safer selection candidates for active surveillance indication and timing of adjuvant treatment	may be of limited availability higher cost
Oncotype DX (Genomic Prostate Score, GPS)	tissue	PCR analysis of 12 cancer related genes and 5 control genes	patients with proven prostate cancer in biopsy	assessment of cancer aggressiveness in low/very low risk patients	safer selection candidates for active surveillance	may be of limited availability higher cost
ARV7	Circulating tumor cells	Detection of ARV7 mRNA in CTC	advanced prostate cancer patients indicated to 2nd generation of antiandrogens	assessment of response to 2nd generation of antiandrogens	optimizing therapy of advanced cancers (e.g. to abirateron/enzalutamide or chemotherapy)	may be of limited availability higher cost
DNA repair genes mutation analysis	tissue	BRCA1/2, ATM, PALB2, CHEK2	castration resistant prostate cancer patients	assessment of response to 2nd generation of antiandrogens or to PARP-inhibitors	optimizing therapy of advanced cancers	may be of limited availability higher cost

ARV7, androgen receptor splice variant 7.

DRE, digital rectal examination.

FISH, fluorescence in situ hybridization.

MiPS, Michigan prostate score.

PHI, prostate health index.

In conclusion, a wide range of diagnostic biomarkers and tests are now available, enabling more precise and personalized prostate cancer management. However, despite these technological advances, test availability and financial considerations remain key factors influencing their integration into routine clinical practice.
